# Can surgically assisted rapid palatal expansion (SARPE) be recommended over orthodontic rapid palatal expansion (ORPE) for girls above the age of 14? A cone-beam CT study on midpalatal suture maturation

**DOI:** 10.1007/s00056-023-00487-x

**Published:** 2023-07-05

**Authors:** Dries Govaerts, Oliver Da Costa, Melisa Garip, François Combes, Reinhilde Jacobs, Constantinus Politis

**Affiliations:** 1 OMFS–IMPATH Research Group, Department of Imaging and Pathology, Faculty of Medicine, Catholic University Leuven, Kapucijnenvoer 33, 3000 Leuven, Belgium; 2 Department of Oral and Maxillofacial Surgery, University Hospitals Leuven, Leuven, Belgium; 3 Department of Oral and Maxillofacial Surgery, AZ Delta Hospital, Roeselare, Belgium; 4 Department of Dental Medicine, Karolinska Institutet, Stockholm, Sweden

**Keywords:** Orthognathic surgery, Cone beam computed tomography, Hypoplasia, Maxilla, Suture, Orthognathe Chirurgie, Digitale Volumentomographie, Hypoplasie, Oberkiefer, Sutur

## Abstract

**Background:**

For patients with a maxillary transversal deficiency (MTD), various treatment options are available, partly based on the practitioner’s experience. This study aimed to determine a cut-off age for decision making between surgically assisted rapid palatal expansion (SARPE) over orthodontic rapid palatal expansion (ORPE) based on skeletal maturation in a female population.

**Methods:**

A total of 100 cone beam computed tomography (CBCT) images of young females were analyzed on maturation of the pterygomaxillary (PMS), zygomaticomaxillary (ZMS), transpalatal (TPS), and midpalatal (MPS) sutures. Based on the maturation of these four junctions, four independent observers had to determine whether they would prefer ORPE or SARPE to widen the maxilla.

**Results:**

For the PMS, the results show a closure of 83–100% from 13 to 17 years. As for the TPS, a closure of 78–85% was observed from 15 years of age. For the 15- to 17-year-old females, a closed ZMS was present in 32–47%. Regarding MPS, closed sutures presented in 61% (stages D and E) of the 15-year-old females. The cut-off age at which SARPE was recommended was 15.1 years for the orthodontist observers and 14.8 years for the maxillofacial surgeon observers.

**Conclusions:**

Significant maturation of MPS was reached at the age of 15 in a female population. The PMS, TPS, MPS, and ZMS closed sequentially. A comprehensive diagnostic approach is necessary for choosing the appropriate treatment. When in doubt, age could assist decision making in a female population, with a cut-off age of 15 years in favor of SARPE based on this study.

**Supplementary Information:**

The online version of this article (10.1007/s00056-023-00487-x) contains supplementary material, which is available to authorized users.

## Abstract (de)

**Hintergrund:**

Für Patienten mit einem transversalen Oberkieferdefizit (MTD) stehen verschiedene Behandlungsoptionen zur Verfügung, die zum Teil auf der Erfahrung des behandelnden Arztes beruhen. Ziel dieser Studie war es, ein Cut-off-Alter für die Entscheidung zwischen einer chirurgisch assistierten schnellen Gaumennahterweiterung (SARPE) und einer kieferorthopädischen schnellen Gaumennahterweiterung (ORPE) auf der Grundlage der skelettalen Reifung in einem weiblichen Kollektiv zu bestimmen.

**Methoden:**

Insgesamt 100 DVT-Aufnahmen von Mädchen wurden hinsichtlich der Reifung der Sutura pterygomaxillaris (PMS), der Sutura zygomaticomaxillaris (ZMS), der Sutura palatina transversa (TPS) und der Sutura palatina mediana (MPS) analysiert. Anhand der Reifung dieser 4 Nahtstellen mussten 4 unabhängige Beobachter entscheiden, ob sie sich eher für eine ORPE oder für eine SARPE zur Oberkieferverbreiterung entscheiden würden.

**Ergebnisse:**

Für die PMS zeigen die Ergebnisse einen Verschluss von 83–100% im Alter von 13–17 Jahren. Bei der TPS wurde ab einem Alter von 15 Jahren ein Verschluss von 78–85% beobachtet. Bei den 15- bis 17-jährigen Frauen lag eine geschlossene ZMS in 32–47% vor. Bei der MPS wurden bei 61% (Stadien D und E) der 15-jährigen Mädchen geschlossene Nähte festgestellt. Das Cut-off-Alter, ab dem eine SARPE empfohlen wurde, lag bei den kieferorthopädischen Beobachtern bei 15,1, bei den Mund-Kiefer-Gesichts-Chirurgen bei 14,8 Jahren.

**Schlussfolgerungen:**

Eine signifikante Reifung der MPS wurde im Alter von 15 Jahren in einem weiblichen Kollektiv erreicht. PMS, TPS, MPS und ZMS schlossen sich aufeinanderfolgend. Für die Wahl der geeigneten Behandlung ist ein umfassender diagnostischer Ansatz erforderlich. Im Zweifelsfall könnte das Alter bei der Entscheidungsfindung in einem weiblichen Kollektiv hilfreich sein, wobei ein Grenzalter von 15 Jahren auf der Grundlage dieser Studie zugunsten einer SARPE ausfällt.

## Introduction

Patients with a maxillary transversal deficiency (MTD) typically present with crowded teeth, buccal corridors, narrowed dental arches, and uni- or bilateral crossbites. To improve the occlusion, maxillary expansion is necessary. Whether orthodontic or surgical maxillary widening is preferred depends on the presumed efficacy of expansion based on the timing of treatment and the practitioner’s experience and training [1].

Orthodontic rapid palatal expansion (ORPE), also known as rapid maxillary expansion (RME), is indicated for the treatment of transversal hypoplasia of the maxilla in young patients. For adolescents and skeletally mature patients, surgically assisted rapid palatal expansion (SARPE), also known as surgically assisted rapid maxillary expansion (SARME), is the gold standard because of the fusion of the sutures [2].

The current literature has a great overlap for the choice between the ORPE and SARPE procedures which prevents a clear cut-off. Depending on the source, the cut-off age recommended for starting SARPE procedures ranges from 14 to 25 years [3–7], while the cut-off age to stop with ORPE procedures ranges from 12 to 25 years [3–7]. Table 1 shows the cut-off for ORPE and SARPE extracted from different articles.

**Table 1:** Cutoff for orthodontic rapid palatal expansion (ORPE) and surgically assisted rapid palatal expansion (SARPE) in published articles Grenzwert für die kieferorthopädische schnelle Gaumennahterweiterung (ORPE) und die chirurgisch unterstützte schnelle Gaumennahterweiterung (SARPE) in veröffentlichten Artikeln

	Max. age for ORPE	Min. age for SARPE
Epker and Wolford [3]	15	16
Timms et al. [4]	25	26
Mossaz et al. [5]	11–20 (second decade)	
Mommaerts [6]	12	14
Alpern et al. [7]	19 (female) and 24 (male)	20 (female) and 25 (male)

*Min.* Minimum, *Max.* Maximum

Most of these statements are based on earlier data derived from skeletal maturation on two-dimensional (2D) imaging and lower resolution techniques. Yet, nowadays, low-dose cone beam computed tomography (CBCT) imaging might help to assist the decision process for or against a surgical intervention. Angelieri et al. [8] suggested classification based on CBCT imaging for a patient-specific assessment before maxillary expansion, with a patient-specific decision based on the maturation of the midpalatal suture (MPS) [9]. Yet, some authors debate on the justified use of CBCT in young patients considering the increased radiation sensitivity of children [10].

In the literature, variable ways of research to determine the maturation of the MPS. Franchi et al. [11] used a CT to check the bone density in Hounsfield units (HU), while ultrasonography via a semi-quantitative bone fill score was used by Sumer et al. [12]. Kwak et al. [13] evaluated the correlation between fractal patterning and ossification of the palatal suture via CBCT in combination with the Angelieri classification. Melsen et al. [14] performed a combined histologic microradiographic study and showed that the transverse growth of the MPS continued up to the age of 16 but argued that interdigitation in adolescence is already so severe that separation of the two halves of the maxilla would not be possible without fracturing the interdigitated processes. They also suggested that the pterygomaxillary suture (PMS) is a possible hinge around which posterior rotation of the maxilla halves occurs as a result of an ORPE treatment [15].

Several studies demonstrated that chronological age is not a good indicator for choosing ORPE vs SARPE since palatal suture fusion is poorly correlated with patient age [16–20]. These studies were solely based on the maturation of the MPS. Isfeld et al. [21] pointed out in their systematic review that the maturity of other maxillary sutures has to be taken into account. Another paper checked the MPS, the zygomaticomaxillary suture (ZMS) and the internasal sutures in an ex vivo study after bone- or dental-borne rapid palatal expansion and identified a high level of strain in the ZMS which emphasizes the complexity of the issue [22]. Kinzinger et al. [23] investigated the MPS as well as the transpalatal suture (TPS) and concluded that the main resistance for expansion lies in the complex connections surrounding the maxilla rather than in the MPS itself. With age, the center of rotation shifts ventrally as a result of the dorsocranially shifting of the resistance, resulting in a V shaped opening in the course of ORPE.

To provide better insight into suture maturation in females (13–17 years of age) and to better assist the decision making for surgical intervention, the present study aimed to determine a cut-off age when to opt for SARPE vs. ORPE based on skeletal maturation in a female population.

## Materials and methods

This study complies with the Declaration of Helsinki “Ethical Principles for Medical Research Involving Human Subjects”. Ethical approval was obtained from the Ethical Review Board of the University Hospitals Leuven (S62686).

A retrospective cohort study was performed on 100 CBCTs of females ranging from 13 to 17 years of age. The images were randomly selected from healthy patients presenting at the Dentomaxillofacial Imaging Center at our hospital from January 2016 to July 2019. All images were obtained for diagnostic indications other than orthodontic decision making for palatal expansion (e.g., impacted canines).

All ages were represented by 20 patients each to create an equilibrium between the age groups. Exclusion criteria were patients with a cleft, a poor-quality scan and previous orthodontic treatment. Only CBCT images of females were included because the patients who came for diagnostic imaging happened to be mostly women. Luckily, they present the largest group in the orthodontic–orthognathic population [24, 25].

CBCT images were acquired according to the following variables: field of view maximum of 230 × 260 to 240 × 190 mm^2^ depending on indication, 96–110 kV, slice thickness 0.3–0.6 mm with a Planmeca Promax 3D Max (Planmeca, Helsinki, Finland) or a NewTom VGi-evo (NewTom, Verona, Italy) scanner and keeping the as low as reasonably achievable (ALARA) principle in mind [10, 26]. Each scan was taken based on a standardized protocol for the positioning as it was suggested by Angelieri et al. with the patient in a natural head position in all three planes (the head in an upright posture, the eyes focused on a point in the distance at eye level, which implies that the visual axis is horizontal) [27].

**Fig. 1:**
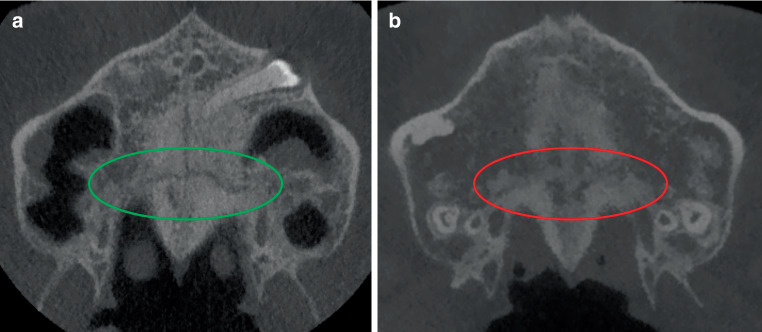
Transpalatal suture: **a** open and **b** closed Sutura palatina transversa: **a** offen und **b** geschlossen

**Fig. 2:**
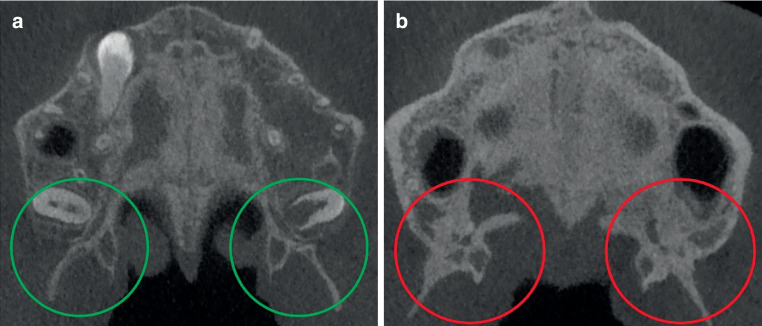
Pterygomaxillary suture: **a** open and **b** closed Sutura pterygomaxillaris: **a** offen und **b** geschlossen

**Fig. 3:**
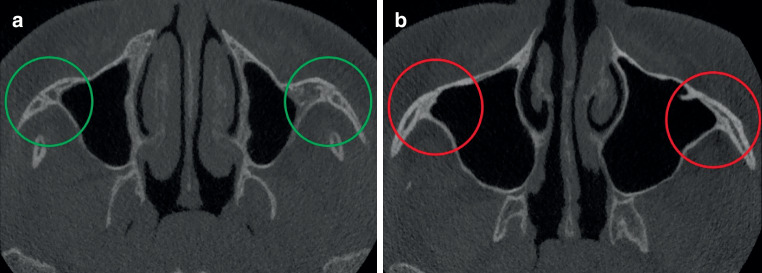
Zygomaticomaxillary suture: **a** open and **b** closed Sutura zygomaticomaxillaris: **a** offen und **b** geschlossen

**Fig. 4:**
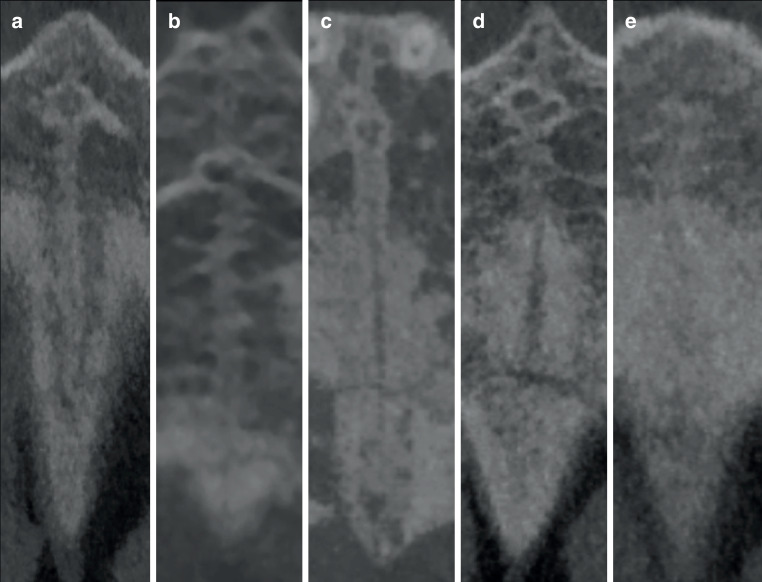
Cone-beam computed tomography (CBCT) images corresponding to the maturation stages by the Angelieri classification [8] of the midpalatal suture (MPS). **a **Stage A: almost straight high-density line; **b **stage B: scalloped high-density line; **c **stage C: two parallel, scalloped high-density lines; **d **stage D: fusion of the MPS only in the palatine bone; **e **stage E: fusion of the MPS in a portion of the maxilla Digitale Volumentomographie (DVT) entsprechend den Reifungsstadien nach der Angelieri-Klassifikation [8] der Sutura palatina mediana (MPS). **a **Stadium A: fast gerade Linie mit hoher Dichte; **b **Stadium B: gewellte Linie mit hoher Dichte; **c **Stadium C: 2 parallele, gewellte Linien mit hoher Dichte; **d **Stadium D: Fusion der MPS nur im Os palatinum; **e **Stadium E: Fusion der MPS in einem Teil des Oberkiefers

Screening of 100 CBCTs was performed randomly and independently by two oral and maxillofacial surgery residents (OMFS) and two orthodontists by assessing the sutures in the maxilla. All CBCT images were anonymized and randomly screened without any knowledge of the patients’ age. The TPS, PMS, and ZMS were analyzed to determine closure, while the level of maturation of the MPS was categorized based on the Angelieri classification [8]. All sutures were assessed in the coronal plane. Figs. 1, 2, and 3 show an open and a closed suture of the TPS, PMS, and ZMS, respectively. The used subdivision of the midpalatal classification is shown in Fig. 4 with exemplary radiographic CBCT images. Stage A appears as an almost straight high-density line, stage B demonstrates a scalloped high-density line, while stage C is characterized as two parallel, scalloped high-density lines. Fusion in the palatine bone of MPS can be visualized in stage D and at stage E, the midpalatal suture is additionally fused in a portion of the maxilla. All stages together represent a gradual transition from an open to a fused suture. Further details on this classification can be found in the study of Angelieri et al. [8]. Based on the results of these four sutures, the observers had also to decide if they would opt for an ORPE or SARPE as the preferred treatment option if the treatment plan would include widening of the maxilla. A subdivision was made based on the training of the examiners (orthodontics or maxillofacial surgery).

A priori sample size analysis (G*Power 3.1, Heinrich-Heine-Universität, Düsseldorf, Germany) was performed based on a similar study [28]. Power analysis calculated that a sample size of minimum of 20 patients per age category would give an 80% probability of identifying a significant difference concerning suture maturation related to patient age at a significance level of 5%. Correlation of suture assessment between the observers was determined using Kendall’s tau (κ). A *p*-value of <0.05 indicated a concordant evaluation of the sutures between the observers.

For assessing the relation between suture evaluation and the suggested intervention, a generalized linear mixed model with patients and observers as random factors and the intervention as fixed factor for binary outcomes with a logit link was fit. For assessing the relation between suture evaluation and age, a similar model with age as fixed continuous factor was fit. For the MPS, the two lowest ranked outcome classes were considered as one group (A–B, C–D) for further outcome analysis. This outcome parameter was associated with age outcome for each observer group. To compare the closure of the left and right sutures for the PMS and ZMS, a Fischer’s exact test was performed. A *P*-value of < 0.05 was considered significant.

To determine a cut-off age for the choice between ORPE and SARPE, a generalized linear mixed model was built between age as a continuous fixed factor and the choice of intervention. Patient and observer were modelled as a random factor. The inflection point of the sigmoidal curve that draws the relation between age and choice was taken as the threshold age.

## Results

The CBCTs of 20 patients per age group ranging from 13 to 17 years were analyzed.

The interrater agreement is presented in Table 2. Both the MPS and TPS suture evaluation showed a significant agreement for the orthodontist (*P* = 0.0059 and* P* < 0.001, respectively) and maxillofacial surgeon (*P* = 0.0032 and* P* < 0.001, respectively) observers. Orthodontists also evaluated the left ZMS with high concordance (*P* = 0.0465). PMS assessment resulted in a nonsignificant correlation both for orthodontist (*P* = 1.00) and maxillofacial surgeon (*P* = 1.00) evaluation.

**Table 2:** Interrater agreement. Kendall’s tau (τ) Interrater-Reliabilität. Kendalls tau (τ)

	OMFS		ORT		Total	
	Kendall’s τ	*P*-value	Kendall’s τ	*P*-value	Kendall’s τ	*P-*value
MPS	0.7154	0.0032*	0.6968	0.0059*	0.5554	< 0.0001*
TPS	0.831	< 0.001*	0.831	< 0.001*	0.831	< 0.001*
PMS Le	0.1557	1	0.2108	1	0.1177	1
PMS Ri	0.15	1	0.2108	1	0.1075	1
ZMS Le	0.2156	1	0.6253	0.0465*	0.2702	0.2736
ZMS Ri	0.2006	1	0.6193	0.054	0.2762	0.2233

*P*-value < 0.05 was considered significant

*OMFS* Oral and maxillofacial surgeons; *ORT* Orthodontist; *MPS* Midpalatal suture; *TPS* Transpalatal suture; *PMS* Pterygomaxillary suture; *ZMS* Zygomaticomaxillary suture; *Le* left; *Ri* right

* Significant values

**Fig. 5:**
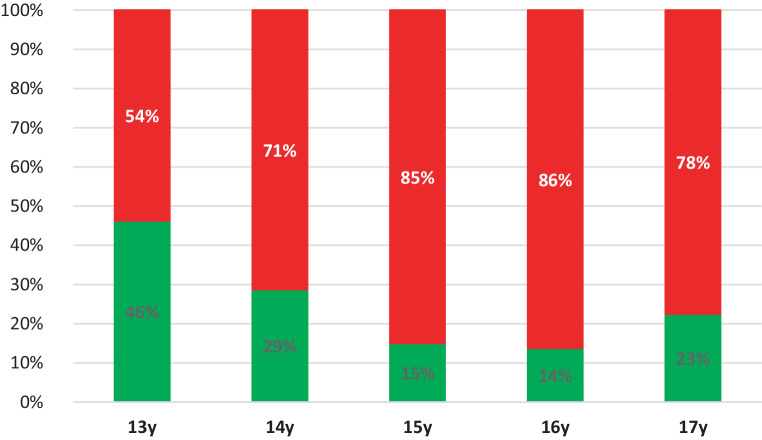
Percentage (%) of transpalatal sutures classified as open (*green*) and closed (*red*), presented per age group. *y* years Prozentsatz (%) der als offen (*grün*) bzw. geschlossen (*rot*) klassifizierten Suturae palatinae transversae entsprechend der Altersgruppe. *y* Jahre

Closure of TPS increased progressively from age 13 to 15 going from 54% to 85%. After this, closure remains stable with 78% to 85%. In Fig. 5, the prevalence of open and closed TPS is shown in percentages per age group, respectively.

**Fig. 6:**
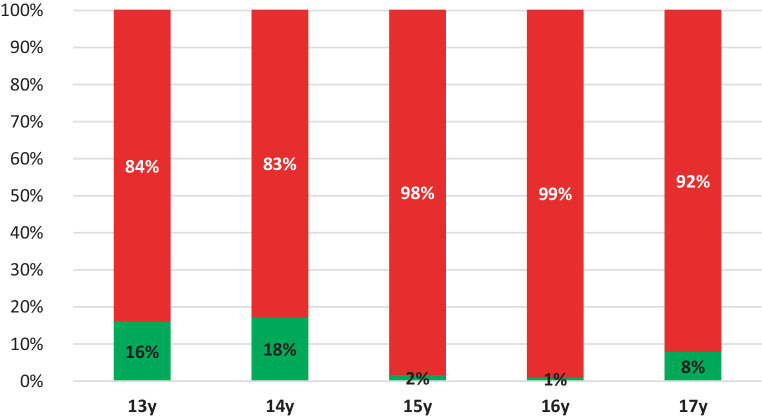
Percentage (%) of pterygomaxillary sutures classified as open (*green*) and closed (*red*), presented per age group.* y* years Prozentsatz (%) der als offen (*grün*) bzw. geschlossen (*rot*) klassifizierten Suturae pterygomaxillares entsprechend der Altersgruppe. *y* Jahre

For the PMS, the results show a closure of 83 to 100% (Fig. 6). The percentages of open and closed PMS are presented per age group, respectively.

**Fig. 7:**
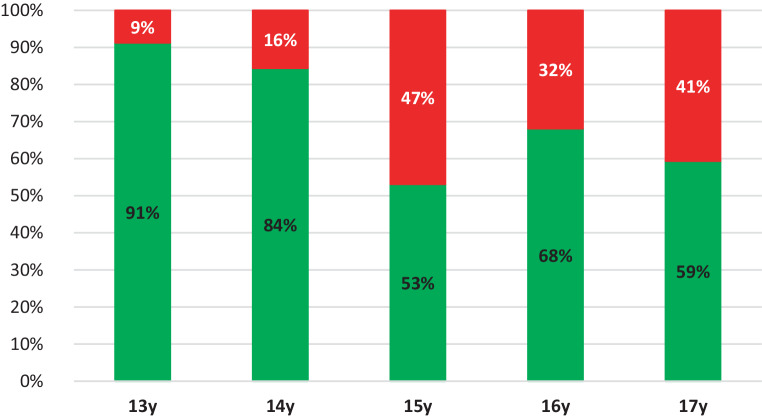
Percentage (%) of zygomaticomaxillary sutures classified as open (*green*) and closed (*red*), presented per age group.* y* years Prozentsatz (%) der als offen (*grün*) bzw. geschlossen (*rot*) klassifizierten Suturae zygomaticomaxillares entsprechend der Altersgruppe. *y* Jahre

The ZMS demonstrated a closure of 9 to 47% depending on the age group. For the 13- and 14-year-old females, a closed ZMS was present in 9 to 16%, while for the 15-, 16- and 17-year-old females a closed ZMS was present in 32 to 47% (Fig. 7).

The Fischer’s exact test to compare the left and right sides of the sutures showed a significant (*p* < 0.001), parallel development of both the PMS and ZMS.

**Fig. 8:**
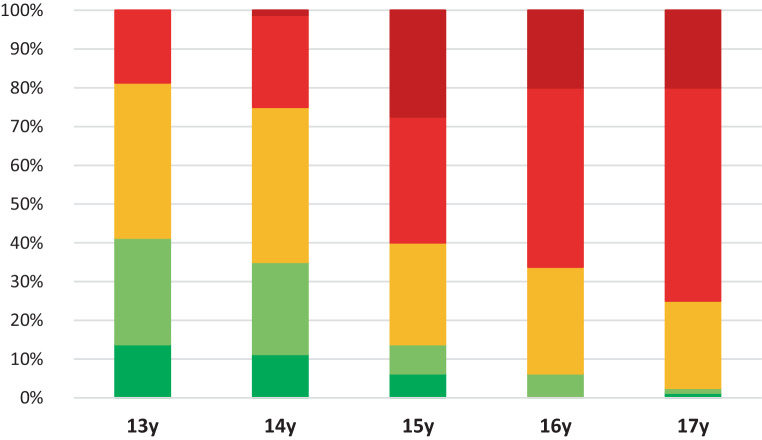
Percentage (%) of midpalatal sutures classified as stage A (*dark green*), B (*light green*), C (*orange*), D (*bright red*), and E (*dark red*), presented per age group.* y* years Prozentsatz (%) der als Stadium A (*dunkelgrün*), B (*hellgrün*), C (*orange*), D (*hellrot*), and E (*dunkelrot*) klassifizierten Suturae palatinae medianae entsprechend der Altersgruppe. *y* Jahre

The average results for the MPS analysis are shown in Fig. 8, where the colors indicate the percentages of stages A–E. The percentages per age group and per stage can be found in supplementary Table 1. The frequency of stages A, B, and C decreased over the age groups as the age increased. A transition was taking place, where stages D and E presented more often with increasing age.

**Fig. 9:**
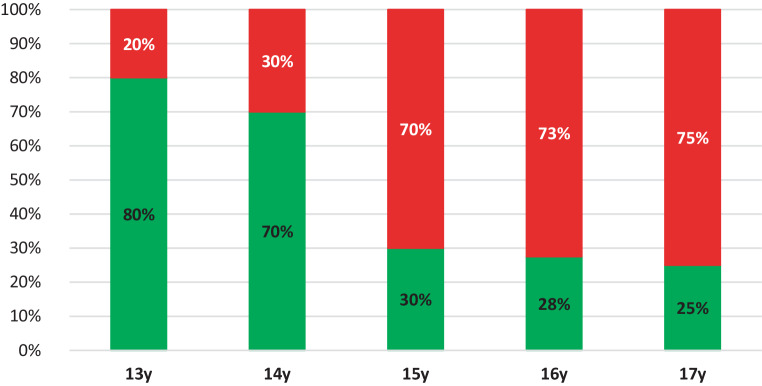
The average of the observers’ indication of treatment between orthodontic rapid palatal expansion (ORPE, *green*) versus surgically assisted rapid palatal expansion (SARPE, *red*) for orthodontists, presented per age group. *y* years Durchschnitt der durch die Beobachter gestellten Behandlungsindikation zwischen kieferorthopädischer schneller Gaumennahterweiterung (ORPE, *grün*) und chirurgisch assistierter schneller Gaumennahterweiterung (SARPE, *rot*) für Kieferorthopäden, dargestellt nach Altersgruppen. *y* Jahre

**Fig. 10:**
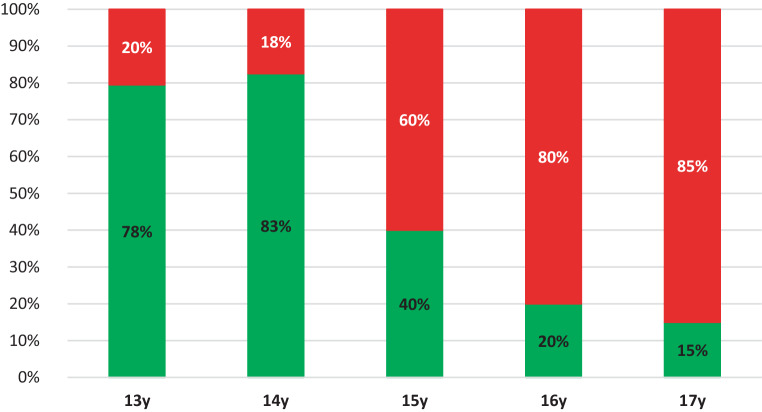
The average of the observers’ indication of treatment between orthodontic rapid palatal expansion (ORPE, *green*) versus surgically assisted rapid palatal expansion (SARPE, *red*) for maxillofacial surgeons, presented per age group. *y* years Durchschnitt der durch die Beobachter gestellten Behandlungsindikation zwischen kieferorthopädischer schneller Gaumennahterweiterung (ORPE, *grün*) und chirurgisch assistierter schneller Gaumennahterweiterung (SARPE, *rot*) für Mund-Kiefer-Gesichts-Chirurgen, dargestellt nach Altersgruppen. *y* Jahre

Table 3 depicts the relation between age and suture evaluation and between suture evaluation and the preference of the doctors for ORPE vs SARPE. These statistical analyses were performed for both orthodontist and maxillofacial surgeon observers. Maxillofacial surgeons reported the MPS and TPS to be significantly correlated with age. Orthodontists reported that MPS, TPS, ZMS, and PMS sutures were significantly more closed with increasing age. Maxillofacial surgeons significantly preferred SARPE intervention with increasing closure of the MPS, TPS, ZMS, and PMS sutures. Orthodontists preferred SARPE intervention when MPS, TPS, and ZMS were more matured. PMS did not significantly influence the orthodontist’s choice for SARPE (*P* = 0.9993). The cut-off age at which SARPE was suggested, based on the evaluation of the sutures, was 15.1 years for the orthodontist observers and 14.8 years for the maxillofacial surgeon observers (Table 4). Figs. 9 and 10 show the average of the observers’ choice of treatment for ORPE versus SARPE for orthodontists and maxillofacial surgeons, respectively.

**Table 3:** Assessment of relation between the evaluation of the maxillary sutures and age of the patient as well as the relation with indication of orthodontic rapid palatal expansion (ORPE) vs for surgically assisted rapid palatal expansion (SARPE). This relation is described for oral maxillofacial surgeons (OMFS), orthodontists (ORT), and both groups (Total). The relation between suture evaluation and intervention was assessed using a generalized linear mixed model with patient and observer as random factors and intervention as fixed factor for binary outcomes with a logit link was fit. For assessing the relation with age, a similar model with age as fixed, continuous, factor was fit Bewertung der Beziehung zwischen der Evaluierung der Oberkiefernähte und dem Alter der Patientin sowie der Beziehung zur Indikation für eine kieferorthopädische schnelle Gaumennahterweiterung (ORPE) vs. für eine chirurgisch assistierte schnelle Gaumennahterweiterung (SARPE). Diese Beziehung wird für Mund‑, Kiefer- und Gesichtschirurgen (OMFS), Kieferorthopäden (ORT) und beide Gruppen (gesamt) beschrieben. Die Beziehung zwischen der Bewertung der Naht und der Intervention wurde anhand eines verallgemeinerten linearen gemischten Modells mit Patientin und Beobachter als Zufallsfaktoren und der Intervention als fixiertem Faktor für binäre Ergebnisse mit einer Logit-Verbindung bewertet. Zur Bewertung des Zusammenhangs mit dem Alter wurde ein ähnliches Modell mit dem Alter als festem, kontinuierlichem Faktor angepasst

		Suture assessment (*P*-value)					
		MPS	TPS	PMS left	PMS right	ZMS left	ZMS right
OMFS	Age	Between 0.0004 and < 0.0001*	0.038*	0.3682	0.1004	0.1384	0.0569
	ORPE vs SARPE	< 0.0001*	0.011*	0.0356*	0.0223*	0.0012*	0.0028*
ORT	Age	Between 0.011* and < 0.0001*	0.019*	0.0017*	0.0017*	< 0.0001*	< 0.0001*
	ORPE vs SARPE	< 0.0001*	0.036*	0.9993	0.9993	0.0014*	0.0014*
Total	Age	0.0034*	0.005*	0.0034*	0.0004*	< 0.0001*	< 0.0001*
	ORPE vs SARPE	0.0002*	0.052	0.0002*	0.0001*	< 0.0001*	< 0.0001*

*P*-value of < 0.05 was considered significant

*MPS* Midpalatal suture; *TPS* Transpalatal suture; *PMS* Pterygomaxillary suture; *ZMS* Zygomaticomaxillary suture

* Significant values

**Table 4:** Surgically assisted rapid palatal expansion (SARPE) preference based on age and observer was determined. A generalized linear mixed model was created between age, as a continuous fixed factor and the choice of intervention. Patient and observer were modelled as random factors. The inflection point of the sigmoidal curve that draws the relation between age and choice was taken as the threshold age Die Präferenz für die chirurgisch unterstützte schnelle Gaumennahterweiterung (SARPE) wurde anhand des Alters und des Beobachters ermittelt. Es wurde ein verallgemeinertes lineares gemischtes Modell zwischen dem Alter als kontinuierlichem festen Faktor und der Wahl des Eingriffs erstellt. Patientin und Beobachter wurden als Zufallsfaktoren modelliert. Der Wendepunkt der sigmoidalen Kurve, die den Zusammenhang zwischen Alter und Entscheidung darstellt, wurde als Altersschwelle angenommen

	OMFS	ORT	Total
Cut-off age (years)	14.8	15.1	14.9

*OMFS* Oral maxillofacial surgeons; *ORT* orthodontists

## Discussion

This study aimed to determine a cut-off age at which significant maturation of the MPS, TPS, ZMS, and PMS is reached, to be used as a key indicator in the decision about which treatment to choose to widen a narrow maxilla: ORPE or SARPE.

From 13 to 15 years, closure of the TPS gradually increased. From the age of 15 years, closure was arrested at about 80% (range 78–85%). On the basis of CBCT studies Kinzinger et al. [23] reported that a complete open TPS was demonstrable only up to the age of 12 years. In cases older than 12 years, only partially open or closed TPS could be identified. They further stated, based on their subgroups, that ORPE occurred in a triangular fashion from 12 years of age onward with a V-shaped change of the palate. It can be argued that this finding should encourage the preference of SARPE if parallel widening is deemed necessary. Thus, when making the choice about treatment, the amount of widening and the shape of widening (parallel or V‑shaped) should be taken into account. The PMS was classified as closed in 83 to 100%. This means that the PMS may be already closed in a large proportion of patients of a younger age than that investigated in this study. Based on CBCT analysis, Ghoneima et al. confirmed that forces provoked by ORPE affected primarily the anterior sutures [29] Thus, one can reason that early closure of the PMS should affect the orthodontic widening of the maxilla. As already indicated, the main point of resistance moves dorsocranial with the development which can compromise ORPE [23]. Thus, our results seem to support the findings of Laudemann et al. [30] that greater posterior expansion is achieved if pterygomaxillary disjunction is performed. However, the systematic review by Hamedi et al. was inconclusive on this topic [31].

In the groups of 13- and 14-year-olds, 84–91% of the ZMS was classified as open bilaterally. Even in the group of 17-year-old females, around 60% of the ZMS was still open. The analysis of CBCTs in this study has shown great heterogeneity in the timing of closure of the different maxillary sutures. It can be concluded that the PMS, TPS, MPS, and ZMS close sequentially and therefore a comprehensive diagnostic approach is necessary for the choice of treatment. The Fischer’s exact test showed a significant result (*p* < 0.001) for PMS and ZMS between the left and right sides. This means that an open left suture is significantly associated with an open right suture. One can conclude that suture closure happens symmetrically.

Maturation of the MPS occurs gradually starting from stage A to stage E with an increase over the age groups. Histological findings of the midpalatal suture support the five maturational stages of the Angelieri classification identified on CBCTs [9]. The prepubertal stages A and B represent a broad suture and the beginning of interdigitation, respectively. The fusion process of the MPS starts in stage C with bone spicules and acellular tissue ‘islands’ [8, 17, 18, 20, 32]. Further maturation takes place in stages D and E with sutural fusion starting in the posterior region and subsequently progresses toward the anterior [16, 17]. Stage C is critical since it is the turning point between the open (A and B) and the closed stages (D and E). In the present study, stages A and B together are represented by 42% and 35% in the age groups of 13 and 14 years, respectively. A turning point is present at the age of 15 years since stages D and E are represented by 61%, 66%, and 75% at the ages of 15, 16, and 17 years, respectively. The orthodontist and maxillofacial surgeon observers suggested SARPE from age 15.1 and 14.8 years; hence, based on our detailed suture maturation analysis, one could state that 15 years is a valuable cut-off age to indicate SARPE.

The answer to the question of the best choice of treatment was based on the comprehensive view of all four sutures (TPS, PMS, ZMS, and MPS). These percentages may be higher than the sutures separately considering the observer’s opinion is based on the combination of all sutures. In this comprehensive approach, the early closure of the PMS obviously pushed the observers to the SARPE procedure, since it is known that fusion of this suture compromises ORPE [1, 15]. It is also proven that the fulcrum of maxillary expansion tends to be located more inferior in adolescents which may be attributed to the increased resistance due to ossification of other maxillary sutures and hereby can adversely affect ORPE treatment [33].

Two biases can be identified for the current study. A selection bias is present because the population selected in this study does not represent patients with MTD. It may be that the MPS in patients with MTD closes earlier or later than in the general population. Thus, further research on this topic is necessary. Furthermore, decision-making is biased by the experience, preference and education of the observer (observer bias). In the present study, we opted to have both maxillofacial surgeons and orthodontists to independently make a decision based on the clinical information and the CBCT images. In practice, the indication for both ORPE and SARPE is most often determined by orthodontists. Adding two OMFSs as observers may have reduced the observer bias but they may have a more surgical preference for treating MTD. In this study, OMFSs were more radical in their decision for ORPE in younger ages and SARPE in older ages. For OMFSs, this may be due to a lack of orthodontic education and the general experience to make direct decisions in everyday practice compared to orthodontists.

This study is further limited by its focus on the indication rather than comparing pre- and postexpansion results. The preference of the observers based on suture maturation on CBCT images is presented instead of actual results postexpansion in patients being treated with an ORPE or SARPE procedure, subdivided into stages of the midpalatal suture maturation. Reliability testing of the classification of Angelieri et al. performed by Isfeld et al. [34] did not match the original study [8]. They stated that the methodology is nonintuitive, requires significant operator calibration, and is heavily influenced by the degree of image sharpness and clarity [34]. A recent study indicated that 81% of the females who were 16 years and older demonstrated a stage D midpalatal suture [35]. This is in line with our results and confirms the idea of the possibility of an age-related treatment choice. This view is also supported by studies that proved that females show less variability in open midpalatal sutures than men and that the midpalatal maturation in females generally occurs earlier than in men [9, 35].

Practitioners should consider all maxillary sutures in the decision-making process regarding ORPE vs SARPE for MTD [21] and also realize that the current classification, presented by Angelieri et al. [8], has its limitations [34]. Nevertheless, in combination with the analysis of all maxillary sutures, the classification is a decent guideline for the choice of treatment between ORPE vs SARPE. It should be remembered that each case is patient-specific. There is the possibility that SARPE may be necessary at an even earlier age, but also that ORPE may be possible in adults [36]. One should also keep in mind the amount of widening as the indication when choosing ORPE over SARPE or vice versa. A CBCT will assist in the decision-making process. However, in the absence of imaging, this study can also provide support in making a treatment choice. More research on the maturation of all maxillary sutures is necessary as this will facilitate the decision of the preferred treatment since other sutures will also affect the postexpansion results.

## Conclusion

Based on cone-beam computed tomography (CBCT) analysis of the maturation of the pterygomaxillary (PMS), transpalatal (TPS), midpalatal (PMS), and zygomaticomaxillary (ZMS) sutures, this study showed that there is a significant maturation of MPS and TPS in a female population of 15 years and older. One could consider 15 years as a possible cut-off age for orthodontic rapid palatal expansion (ORPE) versus surgically assisted rapid palatal expansion (SARPE), but one should keep the amount of widening and indication in mind. When in doubt, individual assessment of circummaxillary suture maturation can be useful for decision-making in ORPE vs SARPE. The classification of Angelieri et al. is a decent guideline in the choice of treatment but all maxillary sutures have to be taken into account.

## Supplementary Information

Supplementary Table 1
