# Supplementary material for: Can surgically assisted rapid palatal expansion (SARPE) be recommended over orthodontic rapid palatal expansion (ORPE) for girls above the age of 14? A cone-beam CT study on midpalatal suture maturation
Source: J Orofac Orthop. 2023 Jul 5;86(1):38–48. doi: 10.1007/s00056-023-00487-x (PMC11746970; doi:10.1007/s00056-023-00487-x)
Supplement: Supplementary file 1 — Supplementary Table 1 [file 56_2023_487_MOESM1_ESM.pdf]

Supplementary Table 1 The percentage (%) per age group and maturation stage of the midpalatal suture (MPS) classified by Angelieri et al. [8]

	**13y**	**14y**	**15y**	**16y**	**17y**
Stage A	14%	11%	6%	0%	1%
Stage B	28%	24%	8%	6%	1%
Stage C	40%	40%	26%	28%	23%
Stage D	19%	24%	33%	46%	55%
Stage E	0%	1%	28%	20%	20%
